# Parenteral Nutrition and Anaphylaxis: A Case Report and Literature Review

**DOI:** 10.7759/cureus.82389

**Published:** 2025-04-16

**Authors:** Otilda M Valderrama V, Stephanie Monteza

**Affiliations:** 1 Department of Surgery, Nutritional Support Committee, Hospital Santo Tomás, Panama, PAN

**Keywords:** allergy and anaphylaxis, complication of treatment, drug-induced anaphylaxis, individualized parenteral nutrition, nutritional therapy, total parenteral nutrition (tpn)

## Abstract

Parenteral nutrition (PN) is a high-risk therapy for patients unable to meet their nutritional needs enterally, carrying risks of hypersensitivity reactions, including anaphylaxis. We present the case of a patient with advanced gastric cancer and a distal subocclusive process that could not tolerate enteral nutrition. PN was initiated, but within five minutes, he developed severe allergic symptoms, which resolved completely after the discontinuation of PN and treatment with antihistamines and steroids. Although hypersensitivity reactions to PN are rare, they can be severe and potentially fatal. Intravenous fat emulsions (IVFEs) and multivitamin solutions are common allergens. This case highlights the need for heightened vigilance during PN administration and the importance of identifying potential allergens. Careful monitoring and individualized care are essential for patients receiving PN. Diagnostic methods are needed to identify specific allergens, prevent future reactions, and improve patient safety.

## Introduction

Parenteral nutrition (PN) involves the intravenous administration of macro- and micronutrients in a complex mixture that can contain up to 40 components, classified as a high-risk medication due to its complexity and potential risks [1,2]. It is indicated for patients who have contraindications to enteral nutrition, those with compromised gastrointestinal tract integrity or function, and those unable to meet their nutritional requirements enterally despite optimization efforts [1].

The numerous components in PN pose a broad potential for hypersensitivity reactions and make identifying specific allergens challenging. However, the prevalence of these reactions is rare, estimated at approximately 1.5 per one million patients receiving PN in the United States [3]. These hypersensitivity reactions can range from mild, self-limiting symptoms to severe, potentially fatal [4]. Their management requires the early identification of symptoms and prompt management [4,5].

We present the case of a patient who experienced a rare but potentially dangerous hypersensitivity reaction to PN and provide a literature review to highlight the need for heightened awareness and vigilance in PN administration, as well as the importance of individualized patient care strategies.

## Case presentation

An adult patient in his mid-50s, without a history of allergies, was admitted to Hospital Santo Tomás in Panama. This is the only highest-level-care hospital operated by the Ministry of Health in this developing country. It is a public adult general hospital with 734 beds, including 30 intensive care unit (ICU) beds, and receives all its funding through state subsidies. As a national referral center, the hospital provides care to a large and diverse patient population, often under resource-constrained conditions.

**Figure 1:**
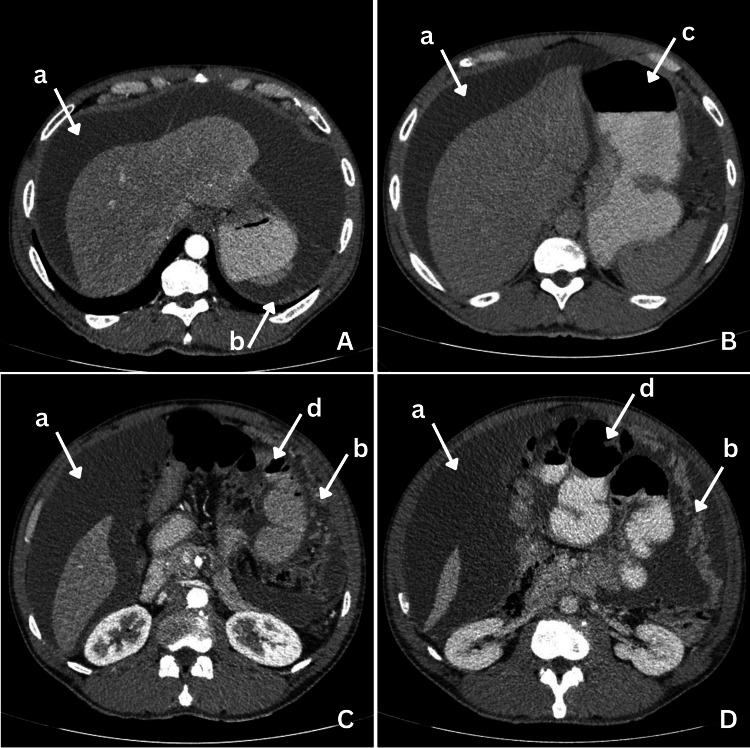
Abdominal CT Scan Abdominal CT scan with oral (A, B, C, and D) and intravenous contrast (A and C). (a) Ascites, (b) images suggestive of carcinomatosis peritoneal, (c) the distension of the gastric chamber and the alteration of the wall, and (d) distended small bowel loops CT: computed tomography

The patient presented with a two-week history of generalized abdominal pain and distention, unquantified weight loss, nausea, and vomiting. Abdominal computed tomography (Figure 1) revealed ascites, the dilatation of the stomach and small bowel loops (suggestive of a subocclusive process), and images compatible with peritoneal carcinomatosis.

**Figure 2:**
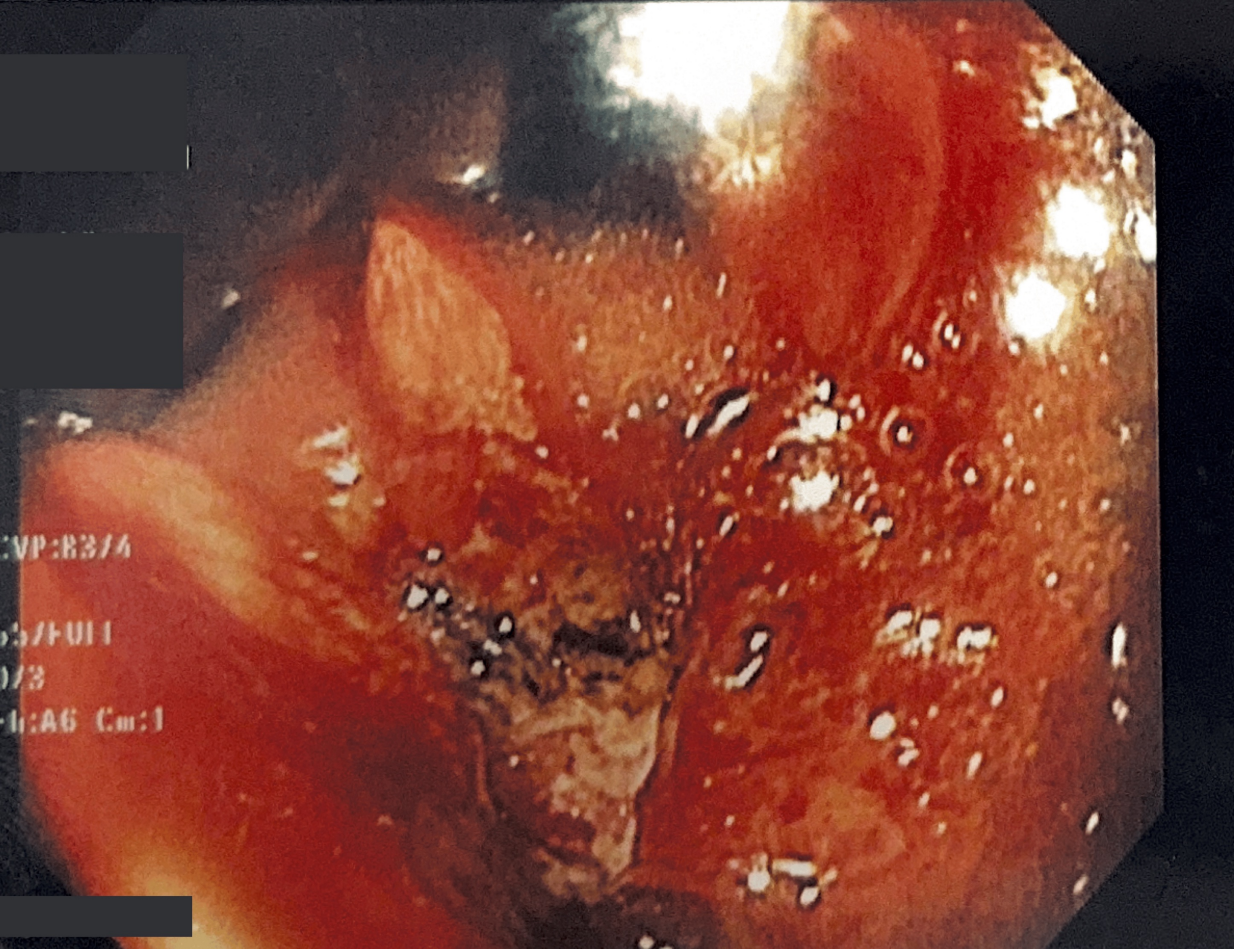
Upper Endoscopy Findings A friable, infiltrating, rigid, and non-distensible lesion of irregular surface

Upper endoscopy showed an infiltrating, rigid, and non-distensible lesion of irregular surface and friable to biopsy, occupying the lesser curvature, posterior wall, and anterior wall of the body, extending from the cardia to the angular incisure (Figure 2). The histopathological report revealed a poorly differentiated carcinoma.

The patient had a 13.6% weight loss, with a current weight of 62.2 kg. Due to the distal subocclusive process, PN was requested. Given the high risk of refeeding syndrome, PN was initiated with 1 g/kg/day of protein, 150 g of carbohydrates, and lipids at 0.4 g/kg/day (15 kcal/kg/day).

PN was initiated at 2:17 pm. At 2:22 pm, the patient reported a sensation of warmth at the catheter site, accompanied by generalized pruritus, respiratory difficulty, angioedema involving the lips and periorbital region, and bronchospasm. He exhibited tachycardia (heart rate of 121 beats per minute {bpm}) and tachypnea (respiratory rate of 22 bpm), with a blood pressure of 116/81 mmHg and an oxygen saturation of 95% on room air. Arterial blood gases revealed mild hypoxemia (partial pressure of oxygen {pO₂} of 70 mmHg) and compensated respiratory alkalosis. PN was immediately discontinued, and treatment was initiated with antihistamines and steroid nebulizations, resulting in the complete resolution of symptoms.

PN was identified as the likely cause, given that the patient had not received any medications or food in the previous eight hours, and the reaction occurred immediately after its administration.

Due to the severity of the reaction and the absence of diagnostic tools in our institution, no allergen-specific tests or skin tests were performed. Moreover, the hospital lacks an allergology specialist, precluding the possibility of a formal allergy consultation.

Considering these limitations and the potential risk of recurrence, a re-challenge with PN, even under antihistamine premedication, was deemed unsafe without appropriate allergological evaluation. However, as the subocclusive process was expected to resolve in the short term, the clinical team opted for a conservative approach: delaying further PN while closely monitoring gastrointestinal recovery. This strategy offered a window of opportunity to transition to enteral feeding, minimizing risk while ensuring that nutritional needs would not be neglected. Had enteral access remained unfeasible, alternative PN formulations would have been considered.

Once the subocclusive process improved, oral nutrition with a hyperproteic and hypercaloric formula was initiated. This approach allowed nutritional requirements to be met with low volumes, which the patient tolerated well.

## Discussion

**Figure 3:**
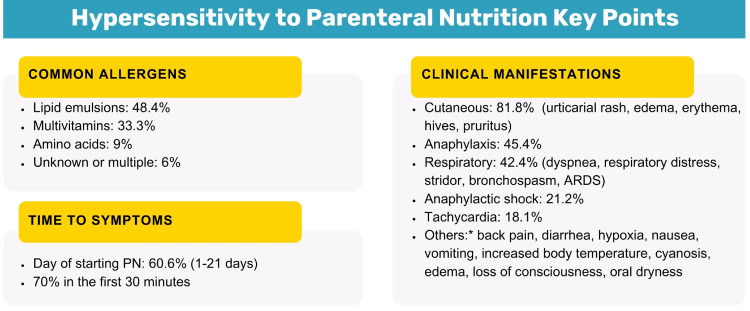
Hypersensitivity to Parenteral Nutrition (PN) Key Points The images are original creations by the authors and created using data/information from the specified source [3] *They presented along with cutaneous and/or respiratory manifestations (exception: diarrhea) ARDS: acute respiratory distress syndrome

The presented case describes a patient with advanced gastric cancer who, due to a distal subocclusive process, could not tolerate enteral nutrition, leading to the initiation of PN. Shortly after, the patient developed a severe hypersensitivity reaction consistent with anaphylaxis. His clinical manifestations align with the literature regarding time to presentation and common clinical manifestations as shown in Figure 3 [3].

The diagnosis of anaphylaxis in adults is clinical and based on established criteria. According to current guidelines, anaphylaxis is highly likely when one of three diagnostic criteria is met: (a) acute onset of symptoms involving the skin, mucosa, or both, plus either respiratory compromise or hypotension; (b) rapid onset of at least two symptoms such as skin-mucosal involvement, respiratory difficulty, hypotension, or gastrointestinal symptoms after exposure to a likely allergen; or (c) isolated hypotension after exposure to a known allergen [4]. In our case, the patient developed generalized pruritus, angioedema, respiratory difficulty, and bronchospasm within minutes of PN initiation, fulfilling the first and second criteria for anaphylaxis diagnosis.

Anaphylaxis is a severe allergic reaction involving multiple systems and can be potentially life-threatening with a lifetime prevalence of 1.6%-5.1% [4,5]. Its onset is rapid and can cause death due to respiratory and/or circulatory compromise, which may occur without typical allergic manifestations [5]. Severe anaphylaxis is associated with older age, preexisting cardiopulmonary diseases, or pharmacological etiology [5].

In patients receiving PN, hypersensitivity reactions can occur across all age groups, mostly within the first half hour of initiation, though cases of up to 21 days later are reported [3]. The most common clinical manifestations are cutaneous, anaphylactic, and respiratory, with 21.2% of patients developing hemodynamic instability [3].

Intravenous fat emulsions (IVFEs) and multivitamin solutions are the most frequently identified allergens [3]. Reactions to IVFEs are not associated with a specific generation, as most contain egg yolk phospholipids or soybean oil [3,6]. Christian et al. highlight that, although IgE-mediated reactions are not usually directed toward lipid molecules, it is thought that the reaction is due to protein contamination from the mentioned sources [3]. Additionally, they noted fewer reactions to IVFE after 2000, likely due to improved manufacturing [3].

Hypersensitivity reactions can occur to any component of PN, including uncommon ones such as trace elements [3,6,7]. In our case, the specific allergen could not be determined due to the lack of specific diagnostic methods in our institution and the severity of the reaction.

Although most patients with hypersensitivity reactions do not have known allergies to PN components, allergic reactions can still occur [3]. Their potential severity is increased by intravenous administration, necessitating careful monitoring [8]. Furthermore, while hypersensitivity reactions often appear within minutes of PN initiation, cases have been reported where reactions develop after several days of uneventful administration [6,9,10]. This underscores the importance of continuous vigilance, as allergic reactions may not be immediately apparent, requiring ongoing assessment throughout PN therapy.

To better understand our case in the context of previously documented hypersensitivity reactions to PN, we compiled a summary of reported cases in the literature (Table 1). This table highlights the variability in patient demographics, the onset of symptoms, suspected allergens, and clinical manifestations. Similar to our patient, many cases involve rapid-onset anaphylactic reactions occurring within minutes of PN initiation.

**Table 1:** Comparison of Hypersensitivity Reactions to Parenteral Nutrition (PN) in Adult Patients in the Literature and Present Case IVFE: intravenous fat emulsion

Study/publication year	Age of the patient (years)	Onset of reaction	Suspected allergen	Clinical manifestations	Management
Connon et al., 1979 [11]	49	Within 24 hours	IVFE (Intralipid®)	Diarrhea	Symptomatic treatment
Faintuch et al., 1981 [9]	58	Day 14	IVFE (Intralipid®)	Diarrhea	IVFE discontinuation
Bass et al., 1984 [12]	68	Within 30 minutes of adding IVFE to PN for the first time	IVFE (Intralipid®)	Anaphylaxis, acute respiratory distress syndrome, cutaneous manifestations, and death	PN discontinuation, supportive care, antihistamines, steroids, and mechanical ventilation
Hiyama et al., 1989 [13]	41	5 hours	IVFE (Intralipid®)	Anaphylaxis, cutaneous manifestations, and respiratory distress	IVFE discontinuation and supportive care
Buchman and Ament, 1991 [14]	36	Day 2	IVFE (Intralipid®)	Cutaneous manifestations	IVFE discontinuation and antihistamines
Andersen and Nissen, 1993 [15]	48	Shortly after	IVFE (Lipofundin®)	Respiratory distress/symptoms, hemodynamic instability, and anaphylaxis	Unknown
Mounier et al., 1995 [16]	30	Few minutes	Multivitamin solution	Anaphylaxis, cutaneous manifestations, and respiratory distress	PN discontinuation, antihistamines, and PN reintroduction without multivitamins
Weidmann et al., 1997 [17]	45, 50, and 51	15 minutes	IVFE (long-chain triglyceride solution)	Cutaneous manifestations, respiratory distress/symptoms, hemodynamic instability, tachycardia, anaphylaxis, and back pain	PN discontinuation, antihistamines, and PN reintroduction with different lipid solutions
Crespí Monjo et al., 2005 [18]	55	Day 1	Multivitamin solution	Cutaneous manifestations	PN discontinuation, antihistamines, and PN reintroduction without multivitamins
Scolapio et al., 2005 [10]	53	Day 16	Multivitamin solution	Cutaneous manifestations	Antihistamines and PN continued without multivitamin
Cragun et al., 2013 [19]	65	Immediately	Multivitamin solution and IVFE	Anaphylaxis, hemodynamic instability, tachycardia, hypoxia, and cutaneous manifestations	PN discontinuation, antihistamines, and PN reintroduction without multivitamins and IVFE
Sánchez Acera et al., 2014 [6]	43	Day 13	Not identified	Angioedema, respiratory distress, and cutaneous manifestations	PN discontinuation, antihistamines, steroids, supportive care, and PN reintroduction with different amino acid solution and without IVFE
Honda et al., 2015 [20]	50	3 days	Amino acid solution (Aminotripa®)	Cutaneous manifestations	PN discontinuation and change of amino acid solution
Çetin et al., 2021 [7]	70	Within two hours	Trace elements	Cutaneous manifestations	PN discontinuation, antihistamines, steroids, and PN reintroduction without trace elements
Present case, 2025	50s	5 minutes	Not identified	Cutaneous manifestation, angioedema, bronchospasm, and anaphylaxis	PN discontinuation, antihistamines, steroids, and transition to enteral feeding

**Figure 4:**
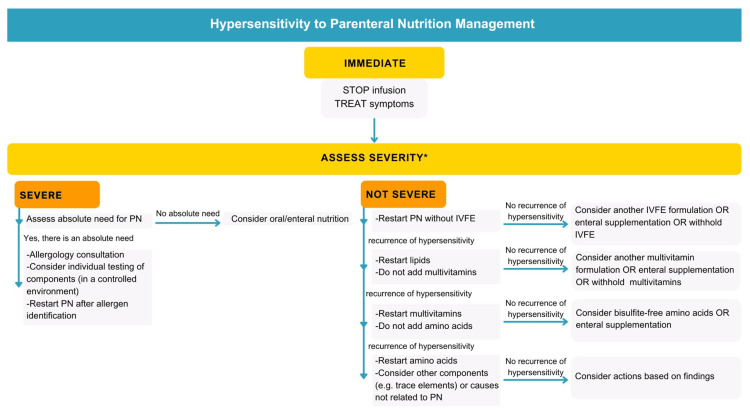
Hypersensitivity to Parenteral Nutrition Management Recommendations The images are original creations by the authors and created using data/information from the specified source [3] *Severe reaction: anaphylaxis or any cardiorespiratory compromise PN, parenteral nutrition; IVFE, intravenous fat emulsion

Management begins with identifying the allergic reaction, stopping PN, and treating symptoms. Christian et al. propose that further management should be based on reaction severity (Figure 4) [3]. For severe reactions, such as anaphylaxis or cardiorespiratory compromise, it is essential to evaluate the absolute need for PN. If necessary, consulting an allergist for the identification and elimination of the allergic component is needed before restarting PN [3].

For non-severe reactions, resuming PN without IVFE is recommended, as it is the most identified allergen [3]. If no hypersensitivity recurrence occurs, a different IVFE can be considered, or PN can continue without lipids, with possible enteral supplementation. If allergic reactions persist, it is recommended to start a systematic approach of therapeutic trials to diagnose the allergen through a process of exclusion, as Figure 4 shows [3].

For patients with egg or soy allergies, few alternatives exist. A 100% fish oil lipid emulsion is effective for soy-allergic patients, while those allergic to eggs should discontinue IVFE and resume enteral feeding as soon as possible [8].

This approach requires close monitoring to detect and treat hypersensitivity reactions early, and successful cases have been reported utilizing this strategy to manage the patient [6].

A significant limitation in our case was the inability to identify the specific allergen due to the absence of specialized diagnostic tests and the unavailability of an allergology specialist. This limited our capacity to confirm the etiology of the reaction and to implement a tailored prevention strategy and safely reintroduce parenteral nutrition. These limitations are not unique to our setting; they reflect common constraints in public healthcare systems in developing countries.

The decision not to re-challenge the patient with PN was based not only on the severity of the initial reaction and limited diagnostic resources but also on the clinical expectation that the subocclusive process would improve. This cautious approach allowed for a safe transition to oral feeding. Nonetheless, contingency plans for nutritional support were in place should the enteral route remain contraindicated, ensuring that nutritional needs would not be compromised.

## Conclusions

Although hypersensitivity reactions to parenteral nutrition are rare, they can be severe and even life-threatening, requiring immediate recognition and management. This case highlights the importance of maintaining a high index of suspicion for allergic reactions in patients receiving parenteral nutrition, even in the absence of a prior allergy history. Given the complexity of parenteral nutrition formulations, careful monitoring during administration is essential to promptly identify adverse events and minimize risks. Additionally, the lack of widely available diagnostic tools in some hospitals to determine specific allergens presents a challenge in clinical practice. Clinicians must adopt an individualized and proactive approach, balancing the benefits of parenteral nutrition with its potential risks, to optimize patient outcomes.
